# Disease severity in hospitalized COVID-19 patients: comparing routine surveillance with cohort data from the LEOSS study in 2020 in Germany

**DOI:** 10.1186/s12879-023-08035-z

**Published:** 2023-02-10

**Authors:** Uwe Koppe, Julia Schilling, Melanie Stecher, Maria Madeleine Rüthrich, Adine Marquis, Michaela Diercke, Martina Haselberger, Carolin E. M. Koll, Michaela Niebank, Bettina Ruehe, Stefan Borgmann, Linus Grabenhenrich, Kerstin Hellwig, Lisa Pilgram, Christoph D. Spinner, Thomas Paerisch, the LEOSS study group, Christoph D. Spinner, Maria Madeleine Rüthrich, Julia Lanznaster, Stefan Borgmann, Kerstin Hellwig, Maria Vehreschild, Christian Hohmann, Frank Hanses, Kai Wille, Bjoern-Erik Jensen, Martin Hower, Siegbert Rieg, Juergen vom Dahl, Jan Rupp, Christoph Roemmele, Nora Isberner, Katja Rothfuss, Lukas Eberwein, Norma Jung, Timm Westhoff, Sebastian Dolff, Richard Strauss, Ingo Voigt, Michael von Bergwelt-Baildon, Uta Merle, Christian Degenhardt, Gernot Beutel, Lorenz Walter, Siri Göpel, Beate Gruener, Dominic Rauschning, Janina Trauth, Milena Milovanovic, Katja de With, Philipp Markart, Jessica Rueddel, Anette Friedrichs, Jan Kielstein, Lukas Tometten, David Heigener, Lars Wojtecki, Joerg Schubert, Wolfgang Guggemos, Stefani Roeseler, Mark Neufang

**Affiliations:** 1 Department of Infectious Disease Epidemiology, Robert Koch-Institute, Berlin, Germany; 2 Department I of Internal Medicine, Faculty of Medicine and University Hospital Cologne, University of Cologne, Cologne, Germany; 3 German Centre for Infection Research (DZIF), Partner Site Bonn-Cologne, Cologne, Germany; 4 Centre for Emergency Medicine, University Hospital Jena, Jena, Germany; 5 Department of Internal Medicine I, Hospital Passau, Passau, Germany; 6 Centre for Biological Threats and Special Pathogens, Robert Koch-Institute, Berlin, Germany; 7 Department of Infectious Diseases and Infection Control, Ingolstadt Hospital, Ingolstadt, Germany; 8 Department for Methods Development, Research Infrastructure and Information Technology, Robert Koch Institute, Berlin, Germany; 9 Department of Neurology, Catholic Hospital Bochum St. Josef-Hospital Bochum, Ruhr University Bochum, Bochum, Germany; 10 Department of Nephrology and Medical Intensive Care, Charité, Universitätsmedizin Berlin, Berlin, Germany; 11 Department of Internal Medicine, Hematology and Oncology, Goethe University, Frankfurt, Frankfurt, Germany; 12 Department of Internal Medicine II, School of Medicine, University Hospital Rechts Der Isar, Technical University of Munich, Munich, Germany

**Keywords:** SARS-CoV-2, COVID-19, Severe COVID, LEOSS, Statutory notification

## Abstract

**Introduction:**

Studies investigating risk factors for severe COVID-19 often lack information on the representativeness of the study population. Here, we investigate factors associated with severe COVID-19 and compare the representativeness of the dataset to the general population.

**Methods:**

We used data from the Lean European Open Survey on SARS-CoV-2 infected patients (LEOSS) of hospitalized COVID-19 patients diagnosed in 2020 in Germany to identify associated factors for severe COVID-19, defined as progressing to a critical disease stage or death. To assess the representativeness, we compared the LEOSS cohort to cases of hospitalized patients in the German statutory notification data of the same time period. Descriptive methods and Poisson regression models were used.

**Results:**

Overall, 6672 hospitalized patients from LEOSS and 132,943 hospitalized cases from the German statutory notification data were included. In LEOSS, patients above 76 years were less likely represented (34.3% vs. 44.1%). Moreover, mortality was lower (14.3% vs. 21.5%) especially among age groups above 66 years. Factors associated with a severe COVID-19 disease course in LEOSS included increasing age, male sex (adjusted risk ratio (aRR) 1.69, 95% confidence interval (CI) 1.53–1.86), prior stem cell transplantation (aRR 2.27, 95% CI 1.53–3.38), and an elevated C-reactive protein at day of diagnosis (aRR 2.30, 95% CI 2.03–2.62).

**Conclusion:**

We identified a broad range of factors associated with severe COVID-19 progression. However, the results may be less applicable for persons above 66 years since they experienced lower mortality in the LEOSS dataset compared to the statutory notification data.

**Supplementary Information:**

The online version contains supplementary material available at 10.1186/s12879-023-08035-z.

## Introduction

The coronavirus disease-19 (COVID-19) pandemic has affected more than 554 million people world-wide until July 2022 [1]. While many patients have only mild or no symptoms, others experience severe disease leading to hospitalization, intensive care treatment, or death [2, 3]. An analysis of health insurance data from 10,021 hospitalized patients from a large statutory health insurance provider in Germany in 2020 showed that 17% of hospitalized COVID-19 patients required mechanical ventilation and 22% died [4]. Another study based on data from 86 German hospitals in 2020 found that 21% (399/1860) of hospitalized COVID-19 patients required intensive care treatment [5].

When patients are newly diagnosed with COVID-19 it can be helpful to identify those at greater risk of disease progression. This will allow to identify vulnerable patients so they can receive appropriate prophylactic treatment to prevent COVID-19 infection or enable early treatment after a COVID-19 diagnosis [6, 7]. In addition, public health practitioners also need to identify populations at greatest risk for severe disease in order to plan appropriate interventions, e.g. vaccination strategies.

Since the beginning of the pandemic, many analyses have been published to identify the greatest risk factors associated with severe COVID-19 disease courses [8]. Age, sex, pre-existing comorbidities, and some laboratory values have been identified as prognostic factors [2, 3, 8, 9]. However, a common question was whether the study population included in the analyses would be representative of the target population [8]. Moreover, these models usually have the best performance in the population where the data was derived from.

Therefore, we analysed clinical data from COVID-19 patients in Germany regarding the risk of severe disease progression and associated factors. In the first section we focused on the representativeness of German patients in the Lean European Open Survey on SARS-CoV‑2 infected patients (LEOSS) [10] comparing this data with German statutory notification data. In the second section we used LEOSS data to identify associated factors of severe disease progression.

## Methods

### Study design

For our analyses, we used data from German patients of the LEOSS study, a cohort study collecting clinical data on COVID-19 patients [10–12]. In addition, we used statutory notification data on hospitalized COVID-19 cases in Germany from the statutory notification system [13] to assess the comparability of the LEOSS data with the COVID-19 patients from the general population.

### Statutory notification data and case selection

Since February 2020 and in line with the German Infection Protection Act, every detection of an infection with SARS-CoV-2 is mandatorily notifiable within 24 h to the responsible local health authority [14]. At the local health authority level, notified cases are validated according to the COVID-19 case definition provided by the Robert Koch-Institute (RKI) and additional case information are investigated [15]. This includes in addition to personal case data and laboratory data (e.g. method, results, date of sampling) information on e.g. vaccination status, symptoms, hospitalization status, risk factors and death. Data fulfilling the case definition are transmitted to the public health offices of the federal states and from there to the RKI. In our dataset, all laboratory confirmed cases (nucleic acid detection, pathogen isolation) reported to the RKI up to 02.06.2021 were included in our analysis focusing on hospitalized cases notified between 01 January 2020 and 31 December 2020.

### LEOSS study design and patient selection

In the LEOSS cohort study, clinicians in participating centers were asked to enter data on COVID-19 patients using an electronic questionnaire on a voluntary basis. The data is stored without any identifiers and an anonymization process is applied to ensure anonymity [16]. It cannot be queried back with data providers at later time points. The cut-off date for data collection was the 11 October 2021.

To be included into the cohort used in this study, patients had to have laboratory-confirmed SARS-CoV-2 infection between 01 January 2020 and 31 December 2020, been hospitalized in a German hospital for at least one day in order to avoid inclusion of outpatients in the dataset, and have complete documentation of their disease stages available. We only included patients from hospitals with at least one patient with a severe disease course. Patients with a SARS-CoV-2 infection were grouped into the following stages of disease severity upon diagnosis: uncomplicated, complicated, critical, or recovered (Additional file 1: Appendix S1).

### Variables

The outcome in this analysis was “severe COVID-19 disease course”, which was defined as either reaching the critical disease stage (defined as at least one of the following: need for catecholamines, life-threatening arrhythmia, mechanical ventilation (invasive or non-invasive) or prolongation of mechanical ventilation, liver failure with Quick < 50%, quick Sequential Organ Failure Assessment (qSOFA) score ≥ 2, renal failure in need of dialysis, Additional file 1: Appendix S1) or death.

We included a broad spectrum of covariables to investigate potential associations with the outcome. All covariables, including laboratory measurements, were recorded at baseline, which included measurements within 48 h after diagnosis. The classification and categorization of the variables was defined through the LEOSS electronic questionnaire [12].

Age was analyzed as a categorical variable with strata of 0–14, 15–25, 26–35, 36–45, 46–55, 56–65, 66–75, 76–85, > 85 years. Sex was investigated as male, female, and diverse. Body mass index (BMI) was analyzed in categories of < 18.5–24.9, 25.0–29.9, 30.0–34.9, and ≥ 35.0 kg/m^2^. The following pre-existing comorbidities were assessed as diagnosed/not diagnosed: respiratory disease (chronic obstructive pulmonary disease (COPD), asthma, other chronic pulmonary disease), cardiovascular disease (hypertension, myocardial infarction, aortic stenosis, atrioventricular block (AV block), carotid arterial disease, chronic heart failure, peripheral vascular disease, atrial fibrillation, coronary artery disease), neurological disease (hemiplegia, dementia, cerebrovascular disease, motoneuron disease, movement disorder, multiple sclerosis, myasthenia gravis, neurological autoimmune diseases), oncological disease (leukemia, lymphoma, solid tumor, metastasized solid tumor, stem cell transplant), diabetes mellitus, connective tissue disease, rheumatic disease, peptic ulcer disease, chronic liver disease, liver cirrhosis, renal disease (chronic kidney disease, acute kidney injury), organ transplantation, and HIV. Requiring dialysis was also analyzed as yes/no. Measures of laboratory values for aspartate transaminase (AST), alanine transaminase (ALT), gamma-glutamyltransferase (GGT), bilirubine, lipase, troponine T, creatinine, urea, lactate dehydrogenase (LDH), D-dimer, and ferritin were analyzed as in the normal range and above the upper limit of normal (ULN) as indicated by the study centers. C-reactive protein (CRP) was analyzed in categories of < 30 mg/l and ≥ 30 mg/l and procalcitonin (PCT) as ≤ 0.5 ng/ml and > 0.5 ng/ml. Lymphocytes were grouped in categories of < 800 cells/µl and ≥ 800 cells/µl, leukocytes as < 4000 cells/µl, 4000–11,999 cells/µl and ≥ 12,000 cells/µl, and neutrophils as < 2000 cells/µl, 2000–8999 cells/µl and ≥ 9000 cells/µl. Symptoms including runny nose, sore throat, dry cough, productive cough, wheezing, dyspnea, palpitations, diarrhea, nausea/emesis, muscle aches, muscle weakness, fever, delirium, excessive tiredness, headache, smell disorder, taste disorder and the absence of symptoms were analyzed either as detected or not detected.

### Statistical methods

The comparison of the LEOSS dataset with the statutory notification data was done using descriptive methods. Continuous variables were reported as medians with interquartile ranges (IQR). Categorical variables were displayed as total numbers and proportions.

To analyze if certain factors were associated with a higher probability of patients to experience the outcome (severe disease course), we used the LEOSS dataset to calculate relative risk estimates (RR) with 95% confidence intervals (CI) using a Poisson regression model with robust standard errors. We performed univariable analyses. Moreover, we performed multivariable analyses adjusted for age and sex since both are well-described factors associated with severe COVID-19 disease.

All analyses were done using Stata, version 17.0 (StataCorp, College Station, Texas 77845 USA).

## Results

Between 01 January 2020 and 31 December 2020, 132,943 hospitalized COVID-19 cases reported to the RKI through the statutory notification system were included (Additional file 1: Appendix S2). Among the COVID-19 cases derived through statutory notification, the first COVID-19 wave from late February to early May 2020 is clearly discernible followed by a period with lower case numbers (Table 1).

**Table 1:** Comparison of hospitalized COVID-19 patients from statutory notifications and the LEOSS cohort, Germany 2020

	Hospitalized cases reported to the RKI in Germany 2020	Hospitalized patients in LEOSS study in Germany
Patients, (n)	132,943	6672
Death of hospitalized cases/patients^1^, % (n)	21.5% (28642)	14.3% (957)
Month of COVID-19 diagnosis, % (n)		
January 2020	0.0% (8)	0.2% (14)
February 2020	0.0% (35)	0.4% (29)
March 2020	7.5% (10,030)	19.7% (1315)
April 2020	11.7% (15,551)	17.0% (1132)
May 2020	2.3% (3100)	3.6% (238)
June 2020	1.0% (1304)	2.0% (132)
July 2020	1.0% (1388)	2.1% (137)
August 2020	1.3% (1783)	1.8% (123)
September 2020	2.0% (2678)	3.0% (197)
October 2020	10.5% (13,896)	13.5% (899)
November 2020	24.6% (32,674)	20.1% (1339)
December 2020	38.0% (50,496)	16.7% (1117)
Age (years), % (n)		
0–14	1.5% (1945)	1.0% (68)
15–25	3.1% (4055)	2.6% (173)
26–35	4.8% (6442)	5.4% (362)
36–45	5.7% (7613)	8.0% (531)
46–55	10.1% (13,417)	13.7% (915)
56–65	14.2% (18,848)	17.6% (1176)
66–75	16.5% (21,914)	17.4% (1158)
76–85	28.8% (38,250)	24.2% (1616)
> 85	15.3% (20,370)	10.1% (673)
Missing	0.1% (89)	–
Sex, % (n)		
Male	51.8% (68,919)	56.6% (3777)
Female	47.9% (63,716)	43.4% (2895)
Diverse	0.0% (8)	–
Missing	0.2% (300)	–
Reported comorbidities, % (n)		
Cardiovascular	24.0% (31,881)	59.8% (3931/6579)
Neurological	6.9% (9163)	24.4% (1509/6172)
Diabetes mellitus	10.2% (13,527)	23.2% (1505/6493)
Pulmonary	7.5% (9956)	15.3% (986/6460)
Cancer	4.8% (6388)	14.5% (938/6453)
Chronic Liver disease	1.3% (1780)	2.1% (134/6417)
Chronic Kidney disease	6.0% (8038)	15.6% (1004/6420)

COVID-19: coronavirus disease 2019, LEOSS: Lean European Open Survey on SARS-CoV-2; ^1^ in LEOSS, only death occurring during the hospital stay has been recorded in the dataset

The second COVID-19 wave started in September 2020 [17], which is also reflected in the increased numbers of reported hospitalized cases (Table 1). In the notification data, majority of hospitalized COVID-19 cases (60.6%) was aged 66 years and above. The distribution of male and female cases was generally balanced. In addition, 21.5% of the notified cases were recorded to have died. The most common reported comorbidity category among notified cases was cardiovascular comorbidities (24.0%), followed by diabetes (10.2%), and pulmonary comorbidities (7.5%).

In the LEOSS cohort, 8051 patients from German hospitals were included. After excluding patients with incomplete or implausible documentation and patients not admitted to the hospital, we obtained a final dataset of 6672 patients (Additional file 1: Appendix S3).

The first COVID-19 wave in early 2020 and the second COVID-19 wave from September 2020 onwards are also clearly observable in the LEOSS dataset (Table 1). However, the proportion of cases included in November (20.1% vs. 24.6%) and December 2020 (16.7% vs. 38.0%) is lower compared to the statutory notification data. In the LEOSS cohort, the proportion of people aged 76–85 years and > 85 years was lower and the proportion of patients aged 36–65 years was higher compared to the cases from the statutory notification (Table 1). In addition, slightly more men (56.6%, 3777/6672) than women (43.4%, 2895/6672) were included in LEOSS.

The proportion of COVID-19 patients who died was overall lower in the LEOSS cohort compared to cases of death among hospitalized cases from the statutory notification data (14.3% vs. 21.5%). Stratified analyses showed that patients up to 65 years had comparable proportions of deceased patients between the datasets while the mortality was lower for patients aged 66 years and older in the LEOSS dataset compared to the statutory notification data (Additional file 1: Appendix S4). Moreover, lower mortality was mostly evident during the months March to May 2020 and October to December 2020, which correlates with the first and second COVID-19 waves in Germany. When analyzing mortality in the LEOSS dataset, we observed that 10.2% (n = 678) of the patients had not recovered at the end of care in the documenting hospital and 60 of those (8.8%) were recorded to have reached a severe disease course. However, it remains unknown if the patients have experienced a severe disease course or death at later stages since the documentation did not allow adding data at a later time point. The proportion of recorded comorbidity categories was higher in the LEOSS patients across all categories with the most common categories being cardiovascular comorbidities (59.8%), neurological comorbidities (24.4%), and diabetes (23.2%).

Among the 6672 hospitalized patients in the LEOSS dataset, 21.4% experienced the outcome of severe COVID-19 disease with 933 reaching the critical disease stage and 497 dying before reaching the critical stage (Table 2).

**Table 2:** Baseline data of COVID-19 patients from the LEOSS cohort

	Overall	Patients without severe disease course	Patients with severe disease course	Crude RR^1^ (95% CI)	Age/Sex-adjusted RR^2^ (95% CI)
Patients, n(%)	6672 (100%)	5242 (78.6%)	1430 (21.4%)		
Follow-up in hospital (days), median (IQR)	8 (4–15)	9 (5–16)	5 (2–11)		
Age (years)					
0–14	68 (1.0%)	63 (1.2%)	5 (0.3%)	0.95 (0.39–2.33)	0.90 (0.37–2.22)
15–25	173 (2.6%)	168 (3.2%)	5 (0.3%)	0.37 (0.15–0.93)	0.41 (0.17–1.03)
26–35	362 (5.4%)	344 (6.6%)	18 (1.3%)	0.64 (0.38–1.10)	0.65 (0.38–1.12)
36–45	531 (8.0%)	490 (9.3%)	41 (2.9%)	1	1
46–55	915 (13.7%)	778 (14.8%)	137 (9.6%)	1.94 (1.39–2.70)	1.85 (1.33–2.58)
56–65	1176 (17.6%)	955 (18.2%)	221 (15.5%)	2.43 (1.77–3.34)	2.34 (1.70–3.21)
66–75	1158 (17.4%)	889 (17.0%)	269 (18.8%)	3.01 (2.20–4.11)	2.94 (2.16–4.02)
76–85	1616 (24.2%)	1153 (22.0%)	463 (32.4%)	3.71 (2.74–5.03)	3.73 (2.76–5.05)
> 85	673 (10.1%)	402 (7.7%)	271 (19.0%)	5.22 (3.83–7.10)	5.65 (4.16–7.69)
Missing	–	–	–		
Sex, n(%)					
Male	3777 (56.6%)	2817 (53.7%)	960 (67.1%)	1.57 (1.42–1.73)	1.69 (1.53–1.86)
Female	2895 (43.4%)	2425 (46.3%)	470 (32.9%)	1	1
Missing	–	–	–		
BMI, n(%)					
< 18.5–24.9	1367 (20.5%)	1077 (20.5%)	290 (20.3%)	1	1
25.0–29.9	1432 (21.5%)	1081 (20.6%)	351 (24.5%)	1.16 (1.01–1.32)	1.15 (1.00–1.31)
30.0–34.9	772 (11.6%)	590 (11.3%)	182 (12.7%)	1.11 (0.94–1.31)	1.20 (1.02–1.41)
≥ 35.0	383 (5.7%)	290 (5.5%)	93 (6.5%)	1.14 (0.93–1.40)	1.42 (1.16–1.74)
Missing	2718 (40.7%)	2204 (42.0%)	514 (35.9%)	–	

^1^Univariable Poisson regression model with robust standard errors; ^2^Multivariable Poisson regression model with robust standard errors adjusting for age and sex. *BMI* body mass index, *IQR* interquartile range

Of the 933 patients reaching the critical stage, 920 had further information on the conditions that marked their transit into this disease stage. Among the clinical markers indicating that a patient had reached the critical stage were septic shock (178/920, 19.3%), qSOFA score ≥ 2 (225/919, 24.5%), congestive heart failure (64/920, 7.0%), life-threatening arrhythmia (99/920, 10.8%), paO2 < 60 mmHg (411/920, 44.7%), mechanical ventilation (561/768, 73.0%), prolongation of mechanical ventilation (114/768, 14.8%), clinical indication for intubation (123/160, 76.9%), severe liver failure (72/920, 7.8%), new renal dialysis (228/920, 24.8%), and any other symptom of the critical stage (81/920, 8.8%).

The probability of reaching the critical disease stage or death increased with age. Being > 85 years was associated with the highest risk for experiencing a severe disease course among hospitalized patients compared to patients between 36 and 45 years (adjusted relative risk (aRR) 5.65, 95% CI 4.16–7.69) (Table 2). In addition, being male was also associated with a higher risk compared to being female (aRR 1.69, 95% CI 1.53–1.86). Having a BMI above 25 was associated with an increased risk of experiencing severe COVID-19 disease with the highest risk among those with an BMI of 35 or higher (aRR 1.42, 95% CI 1.16–1.74) compared to patients with BMI below 25.

A diagnosis of pulmonary comorbidities was associated with an increased risk of severe COVID-19 progression (Table 3). Among those, patients with COPD were at an increased risk (aRR 1.43, 95% CI 1.25–1.63). However, patients with asthma had a lower risk for severe disease (aRR 0.66, 95% CI 0.48–0.89).

**Table 3:** Comorbidities of COVID-19 patients and association with a severe disease progression based on patients from the LEOSS cohort

	Overall	Patients without severe disease course	Patients with severe disease course	Crude RR^1^ (95% CI)	Age/Sex-adjusted RR^2^ (95% CI)
Participants, (n%)	6672 (100%)	5242 (78.6%)	1430 (21.4%)		
Pulmonary comorbidities overall	15.3% (986/6460)	13.8% (703/5096)	20.7% (283/1364)	1.45 (1.30–1.63)	1.29 (1.16–1.44)
COPD	6.9% (442/6423)	5.5% (277/5073)	12.2% (165/1350)	1.88 (1.65–2.15)	1.43 (1.25–1.63)
Asthma	5.1% (327/6422)	5.7% (291/5068)	2.7% (36/1354)	0.51 (0.37–0.70)	0.66 (0.48–0.89)
Other chronic pulmonary disease	4.5% (291/6409)	3.7% (186/5053)	7.7% (105/1356)	1.76 (1.50–2.07)	1.45 (1.24–1.71)
Cardiovascular comorbidities overall	59.8% (3931/6579)	55.3% (2866/5178)	76.0% (1065/1401)	2.14 (1.91–2.39)	1.30 (1.15–1.47)
Hypertension	52.3% (3403/6501)	48.6% (2488/5124)	66.4% (915/1377)	1.80 (1.63–1.99)	1.20 (1.08–1.34)
Myocardial infarction	6.1% (392/6421)	5.0% (256/5072)	10.1% (136/1349)	1.72 (1.49–1.99)	1.25 (1.08–1.44)
Aortic stenosis	2.3% (145/6383)	1.9% (98/5041)	3.5% (47/1342)	1.56 (1.23–1.98)	1.03 (0.81–1.30)
AV block	2.5% (159/6404)	2.0% (102/5062)	4.2% (57/1342)	1.74 (1.41–2.16)	1.18 (0.95–1.47)
Carotid arterial disease	2.2% (143/6373)	1.7% (87/5038)	4.2% (56/1335)	1.91 (1.55–2.35)	1.35 (1.09–1.66)
Chronic heart failure	9.1% (582/6383)	7.1% (359/5048)	16.7% (223/1335)	2.00 (1.78–2.24)	1.41 (1.26–1.59)
Peripheral vascular disease	5.2% (331/6376)	4.6% (231/5042)	7.5% (100/1334)	1.48 (1.25–1.76)	1.03 (0.87–1.22)
Atrial fibrillation	16.0% (1029/6429)	13.7% (696/5074)	24.6% (333/1355)	1.71 (1.54–1.90)	1.14 (1.02–1.27)
Coronary artery disease	15.0% (957/6398)	12.4% (627/5051)	24.5% (330/1347)	1.84 (1.66–2.05)	1.27 (1.14–1.41)
Neurological comorbidities overall	24.4% (1509/6172)	22.3% (1080/4838)	32.2% (429/1334)	1.46 (1.33–1.62)	1.05 (0.95–1.17)
Hemiplegia	1.6% (105/6418)	1.2% (60/5067)	3.3% (45/1351)	2.07 (1.65–2.60)	1.61 (1.28–2.05)
Dementia	9.8% (628/6431)	7.9% (402/5076)	16.7% (226/1355)	1.85 (1.65–2.08)	1.20 (1.06–1.36)
Cerebrovascular disease	9.3% (599/6419)	8.4% (428/5067)	12.6% (171/1352)	1.41 (1.23–1.61)	1.00 (0.87–1.15)
Motoneuron disease	0.1% (5/6110)	0.0% (2/4810)	0.2% (3/1300)	2.82 (1.38–5.79)	1.79 (0.86–3.73)
Movement disorder	2.9% (178/6116)	2.7% (129/4812)	3.8% (49/1304)	1.30 (1.02–1.66)	0.95 (0.75–1.21)
Multiple Sclerosis	0.5% (32/6418)	0.6% (31/5064)	0.1% (1/1354)	0.15 (0.02–1.02)	0.24 (0.03–1.69)
Myasthenia gravis	0.1% (6/6379)	0.0% (1/5031)	0.4% (5/1348)	3.95 (2.76–5.67)	2.63 (1.62–4.26)
Neur. autoimmune diseases	0.2% (15/6380)	0.2% (10/5030)	0.4% (5/1350)	1.58 (0.77–3.23)	1.41 (0.77–2.56)
Oncological comorbidities overall	14.5% (938/6453)	13.2% (674/5091)	19.4% (264/1362)	1.41 (1.26–1.59)	1.15 (1.03–1.29)
Leukemia	1.3% (84/6417)	1.1% (56/5063)	2.1% (28/1354)	1.59 (1.17–2.16)	1.52 (1.11–2.09)
Lymphoma	1.9% (122/6417)	1.6% (81/5065)	3.0% (41/1352)	1.61 (1.25–2.08)	1.38 (1.08–1.76)
Solid tumor	8.9% (573/6431)	8.3% (422/5077)	11.2% (151/1354)	1.28 (1.11–1.48)	0.99 (0.86–1.13)
Solid tumor, metastasized	3.2% (205/6407)	2.8% (142/5061)	4.7% (63/1346)	1.49 (1.20–1.83)	1.38 (1.12–1.69)
Stem cell transplant	0.4% (27/6418)	0.3% (15/5064)	0.9% (12/1354)	2.12 (1.38–3.24)	2.27 (1.53–3.38)
Diabetes	23.2% (1505/6493)	21.0% (1071/5110)	31.4% (434/1383)	1.52 (1.37–1.67)	1.25 (1.13–1.37)
Connective tissue disease	0.4% (25/6415)	0.4% (20/5063)	0.4% (5/1352)	0.95 (0.43–2.08)	1.17 (0.54–2.55)
Rheumatic disease	3.4% (217/6389)	3.2% (162/5037)	4.1% (55/1352)	1.21 (0.96–1.52)	1.13 (0.90–1.42)
Peptic ulcer disease	2.2% (139/6417)	1.9% (96/5064)	3.2% (43/1353)	1.48 (1.15–1.91)	1.20 (0.94–1.54)
Chronic liver disease	2.1% (134/6417)	1.9% (95/5063)	2.9% (39/1354)	1.39 (1.06–1.82)	1.40 (1.08–1.81)
Liver cirrhosis	1.2% (76/6415)	1.0% (51/5065)	1.9% (25/1350)	1.57 (1.14–2.18)	1.50 (1.08–2.07)
Chronic kidney disease	15.6% (1004/6420)	12.7% (645/5073)	26.7% (359/1347)	1.96 (1.77–2.17)	1.44 (1.30–1.59)
On dialysis	3.1% (196/6426)	2.4% (124/5070)	5.3% (72/1356)	1.78 (1.47–2.16)	1.46 (1.21–1.77)
Organ transplantation	1.9% (124/6422)	1.8% (91/5068)	2.4% (33/1354)	1.27 (0.94–1.71)	1.53 (1.14–2.06)
HIV	0.6% (39/6413)	0.6% (32/5064)	0.5% (7/1349)	0.85 (0.44–1.67)	1.13 (0.61–2.11)

^1^Univariable Poisson regression model with robust standard errors; ^2^Multivariable Poisson regression model with robust standard errors adjusting for age, sex at baseline. *BMI* body mass index, *IQR* interquartile range, *Neur* neurological

Moreover, preexisting cardiovascular comorbidities were also associated with an increased risk for severe COVID-19. The highest associated risks were observed among patients with chronic heart failure (aRR 1.41, 95% CI 1.26–1.59), carotid arterial disease (aRR 1.35, 95% CI 1.09–1.66), and coronary artery disease (aRR 1.27, 95% CI 1.14–1.41). Patients with hypertension were also at increased risk for severe disease progression (aRR 1.20, 95% CI 1.08–1.34). Moreover, a previous diagnosis of diabetes (aRR 1.25, 95% CI 1.13–1.37), chronic liver disease (aRR 1.40, 95% CI 1.08–1.81), or liver cirrhosis (aRR 1.50, 95% CI 1.08–2.07) was associated with an increased risk. Similarly, a diagnosis of chronic kidney disease (aRR 1.44, 95% CI 1.30–1.59) or being on dialysis (aRR 1.46, 95% CI 1.21–1.77) was associated with an elevated risk of severe disease progression of COVID-19.

Patients with neurological comorbidities, such as hemiplegia, dementia, and myasthenia gravis were also associated with an increased risk of severe COVID-19 disease progression (Table 3). Regarding oncological comorbidities, a diagnosis of leukemia, lymphoma, stem cell transplant, and metastasized solid tumor was associated with an increased risk. Moreover, having with an organ transplant was also associated with at increased risk.

We did not find evidence for an increased incidence of severe disease courses among patients with connective tissue disease, rheumatic disease, peptic ulcer disease, or HIV (Table 3).

In addition, several blood markers at baseline were associated with an increased risk of severe COVID-19 (Table 4).

**Table 4:** Laboratory values of COVID-19 patients at baseline and association with a severe disease progression based on patients from the LEOSS cohort

	Overall	Patients without severe disease course	Patients with severe disease course	Crude RR^1^ (95% CI)	Multivariable regression RR (95% CI)^2^
Patients, n	6672	5242	1430		
Lab values > ULN, n (%)					
AST	36.8% (1449/3935)	34.0% (1060/3121)	47.8% (389/814)	1.57 (1.39–1.77)	1.54 (1.37–1.73)
ALT	23.4% (1015/4345)	23.0% (787/3429)	24.9% (228/916)	1.09 (0.95–1.24)	1.29 (1.14–1.47)
GGT	38.1% (1539/4042)	35.4% (1129/3188)	48.0% (410/854)	1.50 (1.33–1.69)	1.47 (1.31–1.65)
Bilirubin	6.9% (274/3980)	5.5% (171/3131)	12.1% (103/849)	1.87 (1.58–2.20)	1.55 (1.32–1.82)
Lipase	19.1% (513/2679)	18.5% (395/2132)	21.6% (118/547)	1.16 (0.97–1.39)	1.13 (0.95–1.35)
Troponin T	42.2% (1001/2371)	35.5% (662/1863)	66.7% (339/508)	2.75 (2.33–3.24)	1.95 (1.60–2.38)
Creatinine	31.1% (1524/4906)	25.9% (1002/3865)	50.1% (522/1041)	2.23 (2.01–2.48)	1.67 (1.50–1.86)
Urea	26.9% (1126/4191)	21.1% (702/3327)	49.1% (424/864)	2.62 (2.34–2.94)	1.86 (1.64–2.10)
LDH	62.5% (2687/4297)	58.5% (1972/3372)	77.3% (715/925)	2.04 (1.77–2.35)	1.80 (1.56–2.06)
D-Dimer	68.7% (1855/2701)	64.7% (1370/2119)	83.3% (485/582)	2.28 (1.86–2.79)	1.85 (1.51–2.26)
Ferritin	53.4% (1190/2229)	49.4% (873/1768)	68.8% (317/461)	1.92 (1.61–2.30)	1.74 (1.46–2.07)
CRP ≥ 30 mg/l	51.4% (2522/4907)	45.0% (1738/3859)	74.8% (784/1048)	2.81 (2.47–3.19)	2.30 (2.03–2.62)
PCT > 0.5 ng/ml	14.2% (446/3131)	9.6% (230/2395)	29.3% (216/736)	2.50 (2.21–2.83)	2.21 (1.95–2.50)
Lymphocytes < 800/µl	38.0% (1431/3765)	33.1% (990/2993)	57.1% (441/772)	2.17 (1.91–2.47)	1.79 (1.58–2.03)
Leukocytes < 4000/µl	15.5% (773/4980)	16.0% (630/3932)	13.6% (143/1048)	0.94 (0.80–1.11)	1.05 (0.90–1.22)
Leukocytes ≥ 12,000/µl	9.1% (454/4980)	7.3% (286/3932)	16.0% (168/1048)	1.88 (1.64–2.16)	1.70 (1.49–1.94)
Neutrophils < 2000/µl	13.5% (498/3697)	14.3% (420/2941)	10.3% (78/756)	0.81 (0.65–1.01)	0.93 (0.75–1.15)
Neutrophils ≥ 9000/µl	9.3% (343/3697)	7.3% (216/2941)	16.8% (127/756)	1.92 (1.64–2.25)	1.68 (1.44–1.96)

^1^Univariable Poisson regression model with robust standard errors; ^2^Multivariable Poisson regression model with robust standard errors adjusting for age, sex at baseline. *BMI* body mass index, *IQR* interquartile range, *AST* aspartate transaminase, *ALT* alanine transaminase, *GGT* gamma-glutamyltransferase, *LDH* lactate dehydrogenase, *CRP* c-reactive protein, *PCT* procalcitonin

We found the strongest association with severe COVID-19 for patients with elevated CRP values of 30 mg/l or higher at baseline (aRR 2.30, 95% CI 2.03–2.62), followed by elevated procalcitonin (PCT) > 0.5 ng/ml (aRR 2.21, 95% CI 1.95–2.50), and troponin T above the upper limit of normal (ULN) (aRR 1.95, 95% CI 1.60–2.38). Moreover, elevated liver blood markers (AST, ALT, GGT, bilirubine), and markers of fibrinolysis (D-Dimers) were also associated with a severe disease course (Table 4). While a lymphocyte count of lower than 800 cells/µl was associated with an increased risk of severe COVID-19 (aRR 1.79, 95% CI 1.58–2.03), elevated leukocytes ≥ 12,000/µl (aRR 1.70, 95% CI 1.49–1.94) and elevated neutrophils ≥ 9000/µl (aRR 1.68, 95% CI 1.44–1.96) were also found to be associated. Elevated markers of kidney function, such as creatinine and urea, were also associated with increased risk of severe COVID-19 (Table 4). Similar associations were found for elevated LDH and ferritin at baseline.

Fever was the most commonly reported symptom among hospitalized COVID-19 patients at baseline (47.7%), followed by dry cough (34.1%), and dyspnea (31.6%). In contrast, for 13.5% of patients no signs and symptoms were recorded (Table 5).

**Table 5:** Signs and symptoms of COVID-19 patients at baseline and association with a severe disease progression based on patients from the LEOSS cohort

	Overall	Patients without severe disease course	Patients with severe disease course	Univariable regression RR (95% CI)^1^	Multivariable regression RR (95% CI)^2^
Participants, (n%)	6672	5242	1430		
Asymptomatic	13.5% (875/6475)	15.3% (774/5073)	7.2% (101/1402)	0.50 (0.41–0.60)	0.48 (0.40–0.58)
Runny nose	2.7% (174/6475)	3.3% (165/5073)	0.6% (9/1402)	0.23 (0.12–0.44)	0.32 (0.17–0.60)
Sore throat	5.6% (363/6475)	6.1% (311/5073)	3.7% (52/1402)	0.65 (0.50–0.84)	0.85 (0.66–1.10)
Dry cough	34.1% (2207/6475)	34.5% (1750/5073)	32.6% (457/1402)	0.94 (0.85–1.03)	1.06 (0.96–1.17)
Productive cough	6.3% (409/6475)	6.0% (303/5073)	7.6% (106/1402)	1.21 (1.02–1.44)	1.19 (1.00–1.40)
Wheezing	1.1% (74/6475)	1.0% (53/5073)	1.5% (21/1402)	1.32 (0.91–1.89)	1.26 (0.90–1.75)
Dyspnea	31.6% (2018/6391)	28.4% (1421/5008)	43.2% (597/1383)	1.65 (1.50–1.81)	1.61 (1.47–1.76)
Palpitations	0.9% (58/6475)	0.9% (45/5073)	0.9% (13/1402)	1.04 (0.64–1.68)	1.05 (0.65–1.70)
Diarrhea	9.8% (635/6475)	9.8% (479/5073)	9.8% (138/1402)	1.00 (0.86–1.17)	1.10 (0.95–1.28)
Nausea / Vomiting	9.4% (608/6475)	9.9% (501/5073)	7.6% (107/1402)	0.80 (0.67–0.95)	0.93 (0.78–1.11)
Muscle aches	9.5% (617/6475)	10.3% (525/5073)	6.6% (92/1402)	0.67 (0.55–0.81)	0.88 (0.73–1.07)
Muscle weakness	10.4% (674/6475)	10.3% (520/5073)	11.0% (154/1402)	1.06 (0.92–1.23)	1.04 (0.90–1.19)
Fever	47.7% (3031/6359)	46.0% (2303/5007)	53.8% (728/1352)	1.28 (1.16–1.41)	1.39 (1.27–1.52)
Delirium	1.6% (102/6475)	1.3% (66/5073)	2.6% (36/1402)	1.65 (1.26–2.15)	1.27 (0.98–1.66)
Excessive tiredness	16.2% (1050/6475)	15.8% (803/5073)	17.6% (247/1402)	1.10 (0.98–1.25)	1.06 (0.95–1.19)
Headache	9.1% (591/6475)	10.3% (520/5073)	5.1% (71/1402)	0.53 (0.42–0.66)	0.76 (0.61–0.94)
Smell disorder	4.2% (271/6475)	4.9% (249/5073)	1.6% (22/1402)	0.36 (0.24–0.55)	0.50 (0.33–0.74)
Taste disorder	6.7% (435/6475)	7.7% (391/5073)	3.1% (44/1402)	0.45 (0.34–0.60)	0.57 (0.43–0.75)

^1^Univariable Poisson regression model with robust standard errors; ^2^Multivariable Poisson regression model with robust standard errors adjusting for age, sex and BMI at baseline. *BMI* body mass index, *IQR* interquartile range

Having fever (aRR 1.39, 95% CI 1.27–1.52) and dyspnea (aRR 1.61, 95% CI 1.47–1.76) at baseline were associated with a severe disease course compared to patients without those symptoms. Moreover, we found weak evidence for an association of productive cough with an increased risk of severe disease progression (aRR 1.19, 95% CI 1.00–1.40).

Asymptomatic patients or those, for whom a runny nose, headache, smell disorder or taste disorder was recorded, were less likely to suffer from severe COVID-19 (Table 5).

## Discussion

In our study, we are able to show that 21.4% of the hospitalized COVID-19 patients in Germany included in the LEOSS cohort had a severe course of disease including mechanical ventilation, paO2 < 60 mmHg, septic shock, congestive heart failure, life-threatening arrhythmia, new renal dialysis, or severe liver failure. We found that the LEOSS study population was less representative of the second COVID-19 wave in late 2020 and that it was more likely to represent male patients and patients between 36 and 65 years compared to cases within the statutory notification data. In addition, the proportion of deaths was higher among cases in the statutory notification data compared to the LEOSS cohort especially during the two COVID-19 waves and among patients aged 66 years and older. Conversely, information on comorbidities was more often captured in the LEOSS cohort than in statutory notification data. We identified a broad range of comorbidities, laboratory values and symptoms at baseline that were associated with a higher risk of a severe COVID-19 disease course.

The comparison of the data from the LEOSS cohort with the statutory notification data allows us to assess the comparability of the patients between the datasets. However, both datasets are associated with certain limitations. The statutory notification data in general is supposed to contain personal information on the case (name, address, contact details, age and sex) and test related information (laboratory method, specimen, test result, date of sampling) on all laboratory-confirmed COVID-19 cases. However, further clinical information needs to be inquired by the local health authorities from the patients and/or health care providers. Since the capacities of local health authorities but also of the health care providers might have been severely limited during pandemic waves, we have to assume that the information may be partially incomplete [18, 19]. This is particularly evident when it comes to the recording of comorbidities, which was found at much lower proportions in the notification data compared to the LEOSS cohort and is also corroborated by previous analyses of notification data [19]. While it is possible that the datasets might differ regarding comorbidities, these differences in combination with the higher mortality in the statutory notification dataset make an underreporting of comorbidities in the notification data most plausible. This has also been confirmed by previous analyses of statutory notification data, which reported cases with severe disease more reliably than cases of milder disease [19]. Therefore, we expect the number of hospitalized and deceased cases to be reliable within the notification data. Since age, sex and month of reporting are always recorded in statutory notification data, these variables also seem to be suitable assessing representativeness of the LEOSS dataset.

Regarding the comparability of the LEOSS cases relating to their time of diagnosis we found that the proportion of hospitalized cases in late 2020 was lower compared to those seen in the statutory notification data. This might be attributable to the voluntary participation in LEOSS in a setting of limited resources for documentation during the pandemic. Thus, the dataset might be less representative of patients in the second wave of the pandemic.

In the LEOSS dataset, we found a lower proportion of patients aged 76 years and above compared to the statutory notification data and a higher proportion of patients aged 36–65 years. This could have led to an underestimation of the true proportion of patients with severe disease progression since age is a critical determining factor for prognosis of COVID-19 [2]. This might also explain the substantially higher proportion of deceased patients among cases in the statutory notification dataset compared to the LEOSS cohort. The difference in mortality between the age groups is especially evident among patients aged 66 years and older. One contributing factor to this difference could be that about 10% of the patients in the LEOSS cohort were marked as “not-recovered” at the time of data recording. Since the data in LEOSS are recorded retrospectively after discharge, there might be some patients, especially among the older age groups or those with severe comorbidities, who might have been transferred to end-of-life care or have died at a later time point. Furthermore, medical institutions reporting to LEOSS are predominantly secondary and tertiary care facilities [11], which can offer specialized care and take referrals from primary care hospitals. Thus, very ill patients might deteriorate before they are referred to the hospitals and would not be captured in LEOSS. Moreover, about a third of the residents in Germany of at least 70 years had composed a patient decree [20], in which they would be able to decline invasive ventilation as a treatment option. For these patients the probability to be admitted to a primary care hospital and not be transferred to secondary or tertiary care would be high and they might be underrepresented in the LEOSS dataset. These factors might contribute to the discrepancy between the datasets regarding mortality and consequently to the underestimation of recorded deaths in the LEOSS dataset.

Compared to the statutory notification data, the proportion of men in the LEOSS dataset was slightly higher. Men were shown to have a higher risk for disease progression than women [2], which could have led to an overestimation of the true proportion of patients with severe disease progression. However, since the differences were within a range of 5% between both datasets, we do not expect this to have a major effect on our results.

The effects regarding factors for severe COVID-19 disease progression from our study were generally comparable with the results from previous studies [2, 8, 9]. However, since severe disease progression was not homogenously defined across the studies, the values of the effect estimates can differ. Higher age and male sex were risk factors for severe disease and death in many previous studies including a German study analyzing hospital data as well as in two studies using large national datasets from England and Denmark [3, 5, 21]. In addition, a meta-analysis of 59 studies found that age above 70 years and male sex were associated with an increased risk of severe COVID-19 disease [2].

Similarly, COPD has been described as a risk factor for a severe disease course [9, 22]. While a diagnosis of asthma was not associated with severe COVID-19 in our study, it was associated with an increased risk of death in the OpenSAFELY study in England [3]. Different asthma endotypes and asthma treatments might have a different risk for severe disease courses [23]. Since we do not have further information on the asthma endotypes and treatment in our dataset we are not able to explore the underlying differences that led to these disparate findings.

Hypertension and cardiovascular disease have been described as risk factors for severe COVID-19, which agrees with our study findings [3, 5, 9]. Similarly, the elevated risk associated with diabetes, kidney disease, neurological disorders and patients with organ transplants found in our analysis have been described before [3, 11, 23–26].

CRP has been identified as a good prognostic factor, which we also observed in our analyses [8, 27]. In addition, elevated PCT, troponin T and D-dimers have also been described as prognostic factors [27, 28].

Fever and dyspnea at baseline were associated with severe disease progression in our study and have also been described before in this context [29]. Similar to our findings, a loss of smell or taste has been described before as associated with a less severe disease course [30]. Future studies need to investigate if this relates to a true causal effect or if this association might be related to the fact that in patients with severe disease progression milder symptoms might be less well recorded due to other more severe symptoms.

In an earlier analysis, where LEOSS data were used to estimate risk factors for death among critically ill patients, similar risk factors were identified in both analyses, e.g. underlying cardiovascular and pulmonary diseases [31]. However, some risk factors for severe COVID-19 described here had not been identified in the preceding study, e.g. increased BMI. While this marker appears to be a risk factor to develop a severe course and to be admitted on an ICU, it might be less consequential for the probability of death once a patient had already been admitted to an ICU. Therefore, the results presented here and those of the preceding analyses are complementary to describe the situation of critical COVID-19 disease in the LEOSS population.

### Strengths

A clear strength of our analysis is that we are able to assess the representativeness of the LEOSS cohort using national data from statutory notifications. This allows us to address a common weakness of previous studies where the representativeness of the dataset was often unclear [8]. In addition, we are able to use a large dataset with detailed clinical information for our analyses. Using defined clinical criteria for severe disease progression of COVID-19 is also preferable to using outcomes such as “intensive care treatment” since the indications for intensive care might change throughout the pandemic depending on clinical knowledge and available capacities.

### Limitations

The limitations regarding the completeness of the statutory notification data and the corresponding representativeness of the LEOSS cohort data have been described above. Since the centers participating in the LEOSS study are often secondary and tertiary care centers, the included patients might not be representative of the general population [9]. Moreover, the datasets could only be compared on the distribution of age, sex and mortality so that the comparability regarding other factors could not be assessed. Some symptoms, e.g. excessive tiredness, can be hard to objectively measure across patients and clinics. This might have led to misclassification and could have biased the effect estimate towards the null value. In addition, the data were gathered in 2020 and might be less applicable to SARS-CoV-2 variants that appeared after this time.

## Conclusion

Severe courses of disease progression among hospitalized COVID-19 patients and associated risk factors were comparable to results from previous studies. Comparison of the LEOSS cohort with data from statutory notification revealed that the datasets were comparable regarding the distribution of sex but that the results might be less applicable to patients 66 years and older.

## Supplementary Information

**Additional file 1.** Supplementary data.

## Data Availability

The data from the LEOSS study that support the findings of this study are available from LEOSS Use & Access Committee but restrictions apply to the availability of these data, which were used under license for the current study, and so are not publicly available. Data are however available through the corresponding author upon reasonable request and with permission of the LEOSS Use & Access Committee. The data from the German COVID-19 surveillance system that support the findings of this study are available from the Robert Koch Institute but restrictions apply to the availability of these data, which were used under license for the current study, and so are not publicly available. Data are however available through the corresponding author upon reasonable request and with permission of the Robert Koch Institute.
