# Supplementary material for: Disease severity in hospitalized COVID-19 patients: comparing routine surveillance with cohort data from the LEOSS study in 2020 in Germany
Source: BMC Infect Dis. 2023 Feb 10;23:89. doi: 10.1186/s12879-023-08035-z (PMC9912207; doi:10.1186/s12879-023-08035-z)
Supplement: Supplementary file 1 — Additional file 1. Supplementary data. [file 12879_2023_8035_MOESM1_ESM.docx]

**Disease severity in hospitalized COVID-19 patients: Comparing routine surveillance with cohort data from the LEOSS study in 2020 in Germany**

Supplementary material

Appendix S1 – Definition of disease stages in LEOSS

Appendix S2 – Case selection of hospitalized COVID-19 patients from statutory notification data in Germany in 2020

Appendix S3 - Patient selection of hospitalized COVID-19 patients in Germany in 2020 in the LEOSS study

Appendix S4 - Comparison of mortality between statutory notification and LEOSS data by month and age group

**Appendix S1: Definition of disease stages of COVID-19 patients in the LEOSS study (as described here:**  **https://leoss.net/statistics/)**

| Disease stage | Criteria |
| --- | --- |
| Uncomplicated | asymptomatic or only having symptoms of upper respiratory tract infections, nausea, emesis, diarrhea, or fever |
| Complicated | at least one of the following: need for oxygen supplementation; paO2 at room air<70 mmHg; SO2 at room air <90 mmHg; GOT or GPT >5x upper limit of normal (ULN); new cardiac arrhythmia; new pericardial effusion >1cm; new heart failure with pulmonary edema, congestive hepatopathy or peripheral edema |
| Critical | at least one of the following: need for catecholamines; life-threatening arrhythmia; mechanical ventilation (invasive or non-invasive) and prolongation of mechanical ventilation; liver failure with Quick<50%, qSOFA≥2; renal failure in need of dialysis |
| Recovered | improvement by one degree of severity according to the stages described above and defervescence |

**Appendix S2: Case selection of hospitalized COVID-19 patients from statutory notification data in Germany in 2020**


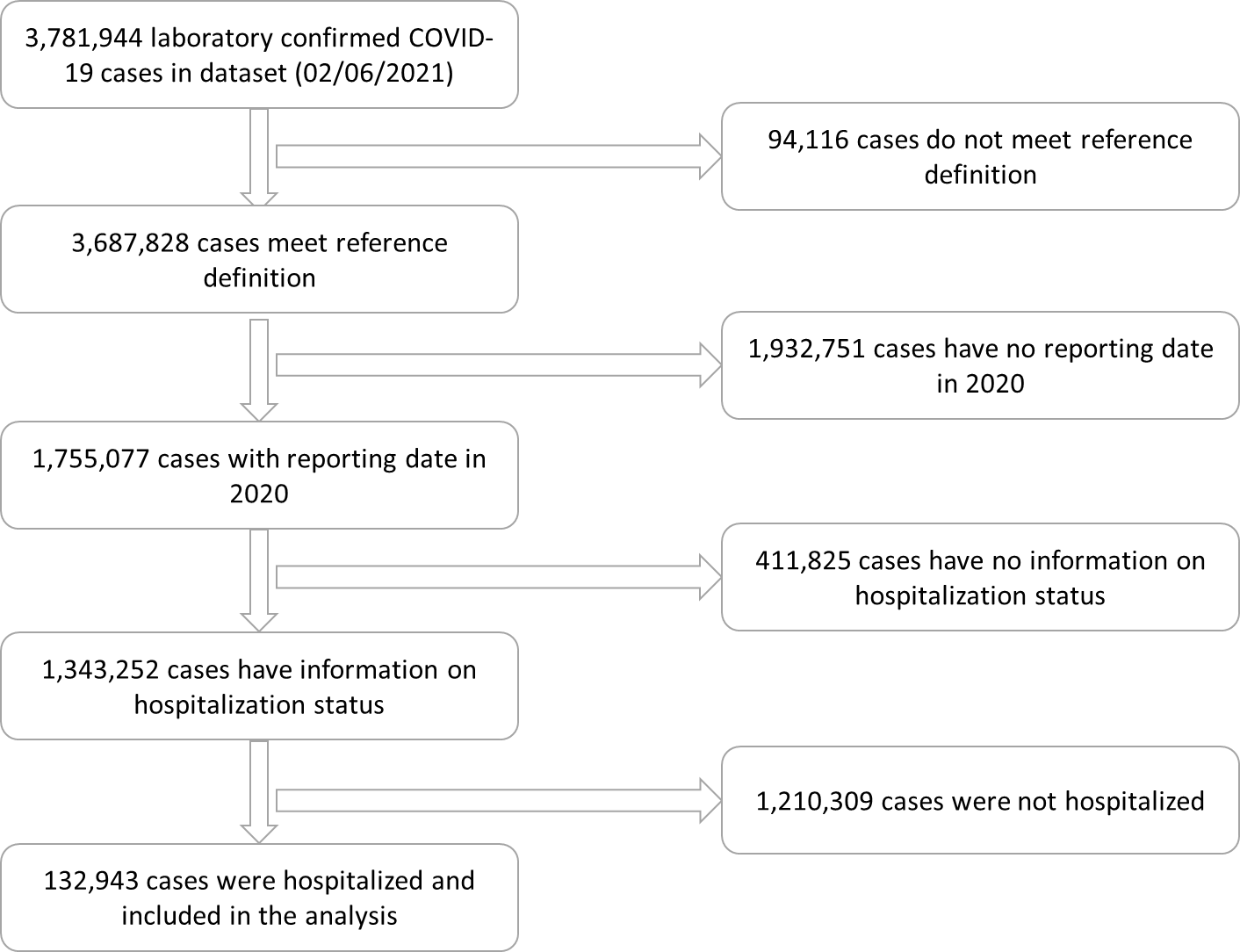


**Appendix S3: Patient selection of hospitalized COVID-19 patients in Germany in 2020 in the LEOSS study**


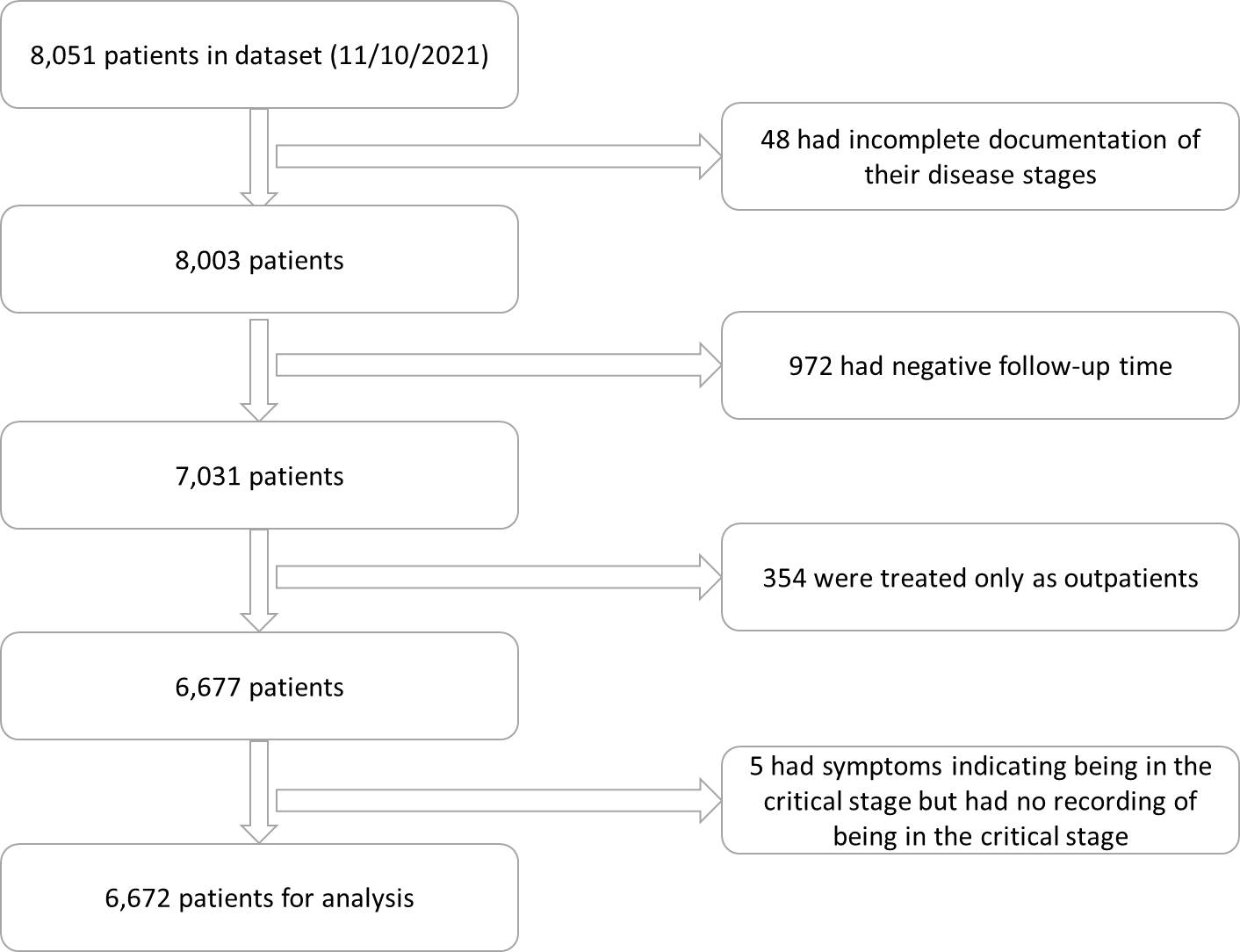


**Appendix S4: Comparison of mortality between statutory notification and LEOSS data by month and age group**

|  | Statutory notification data | | | LEOSS data | | |
| --- | --- | --- | --- | --- | --- | --- |
| Month in 2020 | Overall | Died | Proportion of dead patients | Overall^1^ | Died | Proportion of dead patients |
| January | 8 | 0 | 0.0% | 14 | 2 | 14.3% |
| February | 35 | 1 | 2.9% | 29 | 0 | 0.0% |
| March | 10,030 | 2,149 | 21.4% | 1,315 | 212 | 16.1% |
| April | 15,551 | 3,882 | 25.0% | 1,132 | 206 | 18.2% |
| May | 3,100 | 485 | 15.6% | 238 | 28 | 11.8% |
| June | 1,304 | 116 | 8.9% | 132 | 12 | 9.1% |
| July | 1,388 | 111 | 8.0% | 137 | 9 | 6.6% |
| August | 1,783 | 98 | 5.5% | 123 | 10 | 8.1% |
| September | 2,678 | 246 | 9.2% | 197 | 18 | 9.1% |
| October | 13,896 | 2,009 | 14.5% | 899 | 73 | 8.1% |
| November | 32,674 | 6,639 | 20.3% | 1,339 | 175 | 13.1% |
| December | 50,496 | 12,906 | 25.6% | 1,117 | 212 | 19.0% |

^1^ 26 patients with missing information on vital status were excluded from this analysis

|  | Statutory notification data | | | LEOSS data | | |
| --- | --- | --- | --- | --- | --- | --- |
| Age group (years) | Overall | Died | Proportion of dead patients | Overall^1^ | Died | Proportion of dead patients |
| 0 – 14 | 1,945 | 7 | 0.4% | 67 | 0 | 0.0% |
| 15 – 25 | 4,055 | 6 | 0.1% | 172 | 1 | 0.6% |
| 26 – 35 | 6,442 | 43 | 0.7% | 359 | 3 | 0.8% |
| 36 – 45 | 7,613 | 120 | 1.6% | 530 | 7 | 1.3% |
| 46 – 55 | 13,417 | 546 | 4.1% | 911 | 47 | 5.2% |
| 56 – 65 | 18,848 | 2,027 | 10.8% | 1,169 | 96 | 8.2% |
| 66 – 75 | 21,914 | 4,526 | 20.7% | 1,154 | 169 | 14.6% |
| 76 – 85 | 38,250 | 12,192 | 31.9% | 1,611 | 376 | 23.3% |
| >85 | 20,370 | 9,175 | 45.0% | 673 | 258 | 38.3% |

^1^ 26 patients with missing information on vital status were excluded from this analysis
